# Organic Aluminum Hypophosphite/Graphitic Carbon Nitride Hybrids as Halogen-Free Flame Retardants for Polyamide 6

**DOI:** 10.3390/polym12102323

**Published:** 2020-10-11

**Authors:** Chengxin Guo, Yongshuai Zhao, Guichen Ji, Chaosheng Wang, Zhihan Peng

**Affiliations:** State Key Laboratory for Modification of Chemical Fibers and Polymer Materials, College of Materials Science and Engineering, Donghua University, Shanghai 201620, China; gcx931111@163.com (C.G.); zhaoyongshuai1205@163.com (Y.Z.); jigc1997@163.com (G.J.); cswang@dhu.edu.cn (C.W.)

**Keywords:** graphitic carbon nitride, organic aluminum hypophosphite, halogen-free, flame retardancy, polyamide 6

## Abstract

The novel organic aluminum hypophosphite (ALCPA) and its hybrid (CNALCPA) with graphitic carbon nitride (g-C_3_N_4_) were successfully synthesized and applied as halogen-free flame retardants in polyamide 6 (PA6). Their structures, morphology, thermal stability, and fire properties were characterized. Results showed that both ALCPA and CNALCPA had good flame retardancy. PA6/CNALCPA composites achieved a high limited-oxygen-index (LOI) value of 38.3% and a V-0 rating for UL94 at 20 wt % loading, while PA6/ALCPA composites could reach a V-1 rating for UL94. The flame-retardant mechanism was also studied. On the one hand, the incorporation of g-C_3_N_4_ produced more gas-phase products, which indicated a gas-phase mechanism. On the other hand, g-C_3_N_4_ could catalyze the thermal degradation of ALCPA and PA6 to form a compact char layer that was evidence for a solid-phase mechanism. The tensile test of the PA6 composites also displayed good mechanical properties.

## 1. Introduction

Polyamide 6 (PA6), a light and nontoxic material, is widely used in many fields due to its excellent properties of toughness, corrosion resistance, and processability [1]. However, flammability and the droplet phenomenon during combustion limit its application [2,3].

A common method in improving the flame retardancy of PA6is to add flame retardants [4]. Halogen flame retardants, especially bromine-containing flame retardant (BFR), are one of the most important types for PA6. BFR has high flame-retardant efficiency, good thermal stability, and little effect on the mechanical properties of the materials. However, BFR generates toxic gas (hydrogen bromide) when burning, which pollutes the environment and seriously threatens human health [5,6].

In recent years, the development of halogen-free flame retardants, such as phosphorus-based ones has become a hot topic [7]. Cerium hypophosphite (CeHP) was synthesized by Tang et al., and it is used in glass-fiber-reinforced PA6 [8]. The results showed that the limited-oxygen-index (LOI) value of flame-retardant composites containing 20 wt % CeHP was 26.5%. Heat-release rate and total heat release were reduced by 27.1% and 21.1%, while tensile strength was increased by 25%. A UL94 V-0 rating was also confirmed with the vertical burning test. Two phosphorus–nitrogen flame retardants were synthesized by Tao et al. [9] using chlodiphenylphosphine (CDP) or diphenylphosphinyl chloride (DPP) and cytosine (Cy), and CCDP and CDPP had a good synergistic effect. With the addition of 8 wt % CDPP, the vertical burning rating of the composites reached V-0, and the LOI value was 31.1%.

Graphitic carbon nitride (g-C_3_N_4_) is the most stable carbon nitride allotrope [10]. Atomic layers are formed by covalent bonds between C–N atoms and they stack with each other [11]. The compound g-C_3_N_4_ has attracted more attention because of its metalfree photocatalytic ability [12,13]. By loading inorganic materials or other methods, it is possible to impart different properties to g-C_3_N_4_ and expand its application area [14,15,16,17]. In addition, the triazine ring and high thermal stability of g-C_3_N_4_ make it a good candidate for flame retardancy.

Shi et al. [18] studied flame-retardant applications of g-C_3_N_4_. G-C_3_N_4_ was first modified with phosphinic acid oligomers, and two kinds of g-C_3_N_4_/organic aluminum hypophosphite (OAHPi) hybrids were synthesized and incorporated into polystyrene (PS). It was anticipated that these hybrids were effective in enhancing the fire safety of PS and could be used as good smoke suppressants. Zhang et al. [19] succeeded in synthesizing a core-shell g-C_3_N_4_/PAZn flame retardant by self-assembly and using it in epoxy resin (EP). Cone-calorimetry results indicated that g-C_3_N_4_/PAZn-EP improved the fire safety of EP composites, and functioned well as a flame retardant and smoke suppressant.

In this work, the novel organic aluminum hypophosphite (aluminum 2-carboxyethylphenylhypophosphite, ALCPA) and its hybrid (CNALCPA) with g-C_3_N_4_ were simply synthesized through a salification and hybridation reaction, and applied in PA6 to measure its flame retardancy. The thermal properties and flame retardancy of PA6 composites were investigated, and the mechanism for PA6 fire-hazard reduction is proposed. ALCPA and CNALCPA provide valuable references about phosphorus flame retardants and expand the application of PA6.

## 2. Materials and Methods

### 2.1. Materials

2-Carboxyethylphenyl phosphoric acid (CEPPA, 99.0%) was provided by Changxing New Chemical Material Development Co., Ltd. (Dezhou, China). Aluminum sulfate (Al_2_(SO_4_)_3_·18H_2_O, 99.7%), melamine (MEL, 99.7%), and sodium hydroxide (NaOH, 99.7%) were purchased from Sinopharm Chemical Reagent Co., Ltd. (Shanghai, China). PA6 (BL3280H, relative viscosity is 2.8) was supplied from Baling Petrochemical Corporation Branch (Yueyang, China). Deionized water was obtained through a thermal-purification system. All chemical agents were used without further treatment.

### 2.2. Synthesis of g-C_3_N_4_

G-C_3_N_4_ was prepared on the basis of reported work [20]. The brief process was as follows. First, MEL was heated at 450, 550, and 650 °C for 2 h with a heating rate of 10 °C/min. Second, the light-yellow product was washed with deionized water to remove unreacted MEL. Lastly, the purified product was mechanically crushed after drying at 120 °C for 4 h.

### 2.3. Synthesis of ALCPA

ALCPA was synthesized through salification. First, 0.1 mol CEPPA, 0.2 mol NaOH, and 300 mL of deionized water were mixed in a 500 mL three-neck flask under stirring. Then, 60 mL of 1 M aluminum sulfate solution was added dropwise into the flask. Stirring was maintained for 4 h to complete the reaction at 80 °C. After that, the precipitate was filtered, washed with deionized water, and dried at 80 °C overnight.

### 2.4. Synthesis of CNALCPA

**Scheme 1:**
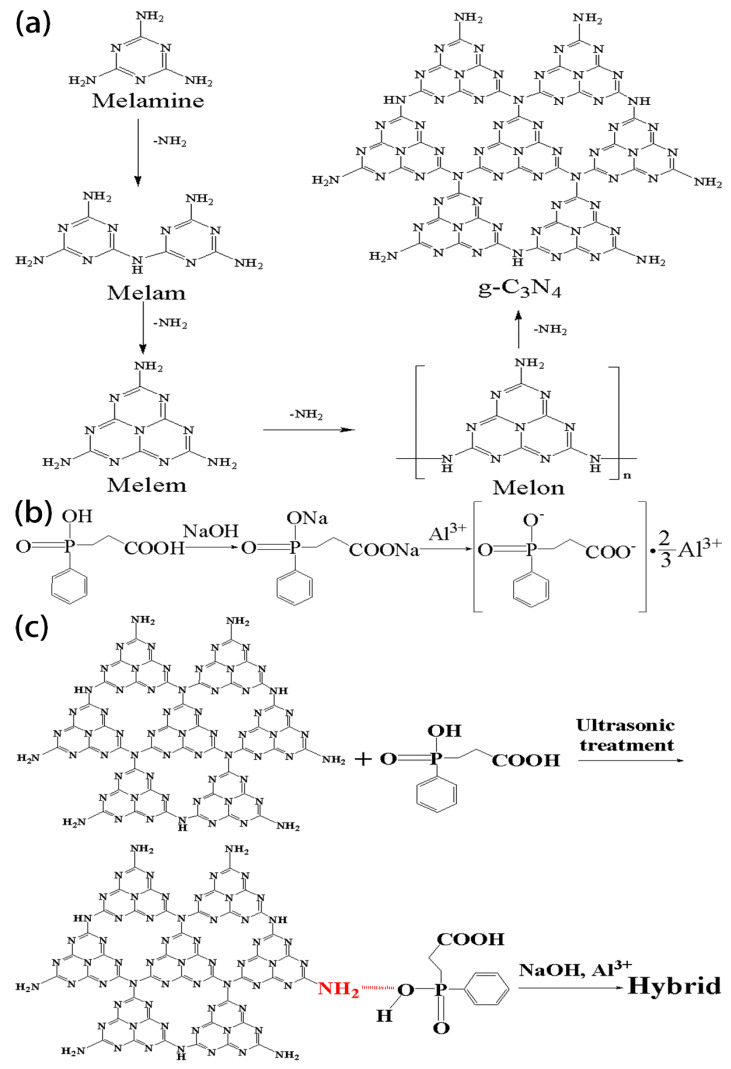
Formation mechanism of (**a**) graphitic carbon nitride (g-C_3_N_4_), (**b**) aluminum 2-carboxyethylphenylhypophosphite (ALCPA), and (**c**) hybrid ALCPA (CNALCPA).

The synthesis of CNALCPA was similar to that of ALCPA. First, 1.15 g g-C_3_N_4_ and 0.1 mol CEPPA were dispersed in 300 mL deionized water with stirring and ultrasonically treated for 1 h at 80 °C. Then, 0.2 mol NaOH was added. After stirring for 30 min, 60 mL of 1 M aluminum sulfate solution was dropped slowly into the system. White precipitate was generated and filtered, washed, and dried. This hybrid was denoted as CNALCPA5. Furthermore, CNALCPA10, CNALCPA20, or CNALCPA30 was prepared by the same approach, where 10, 20, and 30 refer to the weight ratio of g-C_3_N_4_ to ALCPA. The formation mechanisms of g-C_3_N_4_, ALCPA, and CNALCPA are illustrated in Scheme 1.

### 2.5. Preparation of PA6 Composites

PA6 composites were prepared by a typical method. ALCPA and CNALCPA were added into PA6 at 240 °C using a screw extruder (HKY-35, Nanjing Ruiya polymer Equipment Co., Ltd., Nanjing, China, screw speed: 250 r/min, screw temperature: 240 °C). The obtained particles were subsequently made into standard shapes by injection moulding (MB108T, Jinsheng Plastic Machinery Co., Ltd., Nanjing, China, moulding temperature: 260 °C, moulding pressure: 100 bar). PA6 containing ALCPA was labeled as PA6/ALCPAχ, where χ was 5, 10, 15, or 20 for added 5, 10, 15, or 20 wt % of ALCPA, respectively. The same procedure was employed to obtain samples containing CNALCPA5, CNALCPA10, CNALCPA20, or CNALCPA30, and the addition amount for all was 20 wt %. For comparison, pure PA6 was also fabricated. The composition of PA6 composites is shown in Table 1. Each sample was manufactured to be at least 2 kg.

### 2.6. Characterization

Fourier transform infrared spectra (FTIR, Nicolet 6700, Thermo Fisher, Waltham, MA, USA) were established to investigate the functional groups. Each sample was scanned 32 times from 4000 to 600 cm^−1^ with a resolution of 2 cm^−1^. X-ray diffraction patterns (XRD, D/max-2550 PC, Rigaku, Tokyo, Japan) were provided to determine the crystal structure with a scanning range from 5° to 90°. The contents of each element were tested by atomic-emission spectra (AES, Vario EL III, Elementar, Langenselbold, Germany). Molecular structures were studied by ^1^H nuclear magnetic resonance (^1^H-NMR, NAVANCE400, Bruker, Karlsruhe, Germany) at room temperature with heavy water (D_2_O) as the solvent. The morphology of materials and char residue was recorded using a scanning electron microscope (SEM, S-4800, Hitachi, Japan). Sample thermal stability was established through thermogravimetric analysis and differential thermogravimetric analysis (TGA and DTG, TG 209 F1, Netzsch, Selb, Germany) from 50 to 750 °C with a heating rate of 10 °C/min under an N_2_ atmosphere. The fire properties of the composites were measured with the limited-oxygen-index test according to ASTM D2863-97 (LOI, JF-3, Nanjing Jionglei Instrument Co., Ltd., Nanjing, China), vertical burning test according to ASTM D3801 (5402, Suzhou Yangshuowoerqi Detection Technology Co., Ltd., Suzhou, China), and cone-calorimeter test according to ISO5660-1 (6810, Suzhou Yangshuowoerqi Detection Technology Co., Ltd., Suzhou, China). The instrument radiant power of the cone calorimeter was 50 kW/m^2^. Tensile strength was analyzed with an electronic universal testing machine (WDW3020, Changchun Kexin Instrument Co., Ltd., Changchun, China) with a tensile rate of 50 mm/min according to ASTM D-638. An average of at least five individual determinations were obtained. The dimensions of the samples for LOI, vertical burning, cone calorimeter and tensile test were 120 × 6.5 × 3.2 mm^3^, 130 × 12.5 × 1.6 mm^3^ (thin)/130 × 12.5 × 3.2 mm^3^ (thick), 10 × 10 × 3.0 mm^3^ and 120 × 13 × 3.5 mm^3^ respectively. Differential scanning calorimeter (DSC) was performed by Diamond DSC (PerkinElmer, Waltham, MA, USA), and the heating rate was 10 °C/min under N_2_ atmosphere.

## 3. Results and Discussion

### 3.1. Characterization of ALCPA and CNALCPA

**Figure 1:**
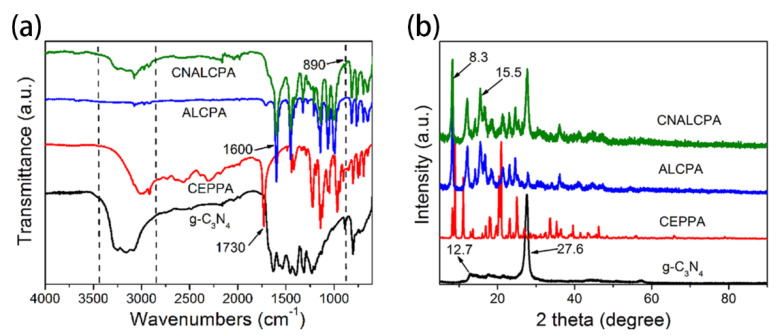
(**a**) FTIR spectra and (**b**) XRD patterns of g-C_3_N_4_, 2-carboxyethylphenyl phosphoric acid (CEPPA), ALCPA, and CNALCPA.

The FTIR technique was adopted to investigate the functional groups of g-C_3_N_4_, CEPPA, ALCPA, and CNALCPA, as shown in Figure 1a. The broad absorption band at 2800~3400 cm^−1^ of g-C_3_N_4_ was derived from the remaining primary and secondary amine groups of the incomplete thermal condensation. The peak at 890 cm^−1^ was caused by the substitution of triazine ring [21]. The absorption peak at 805 cm^−1^ was due to the bend vibration of the triazine ring, and the band at 1700~1000 cm^−1^ was attributed to the stretching vibration of the connected units. These major absorption peaks could be clearly observed in CNALCPA. In addition, the most significant peak in CEPPA was the –C=O group appearing at 1730 cm^−1^. However, in ALCPA, because of the salification reaction, –COOH disappeared and turned into –COO^−^. Therefore, the characteristic peak of carboxylates that appeared at 1600 cm^−1^ became reasonable. This feature was also demonstrated in CNALCPA.

XRD measurement was employed to study the crystal phase of the materials. As shown in Figure 1b, there were two distinct diffraction peaks of g-C_3_N_4_ at 12.7° and 27.6°. The diffraction peak at 12.7° was caused by a {001} crystal face, while the peak at 27.1° was due to a {002} crystal face. These two diffraction peaks were in good agreement with the data on PDF#50-1512. The obvious peaks of ALCPA were at 15.5° and 8.3°. These diffraction peaks were totally detected in CNALCPA when g-C_3_N_4_ was combined with ALCPA.

Atomic-emission spectra and ^1^H nuclear magnetic resonance were used to prove the successful synthesis of ALCPA. The comparison of the theoretical and the actual contents of each ALCPA element is listed in Table 2. The actual contents of ALCPA were only slightly different from the theoretical ones. These differences were within the tolerance range of the instrument.

**Figure 2:**
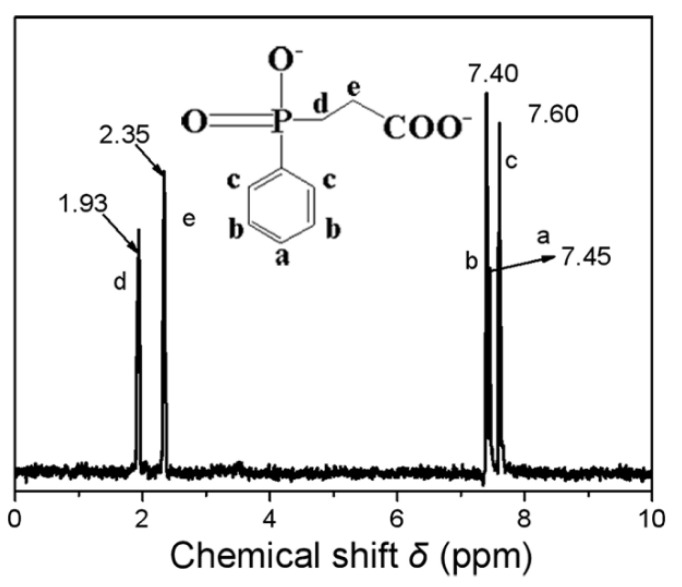
^1^H-NMR spectrum of ALCPA.

With regard to the ^1^H-NMR spectrum of ALCPA, shown in Figure 2, the multiple peaks appearing at 7.4, 7.45, and 7.6 were proton peaks of the benzene ring. The rest of the peaks (δ = 1.93 and 2.35) were attributed to the alkyl group. Along with FTIR and XRD analysis, the successful synthesis of ALCPA was confirmed again.

**Figure 3:**
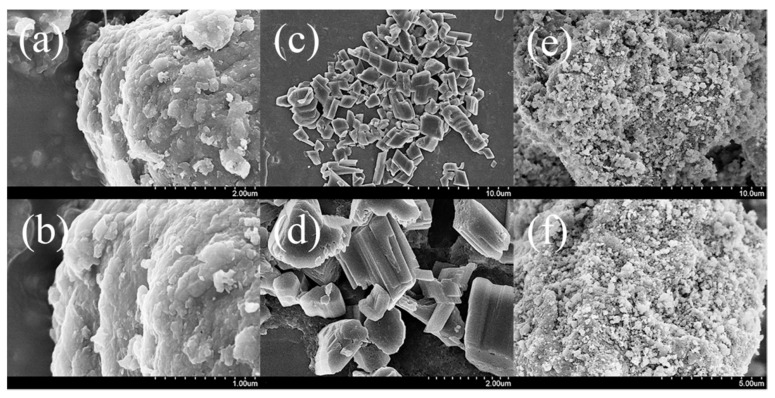
SEM images of (**a**,**b**) g-C_3_N_4_, (**c**,**d**) ALCPA, and (**e**,**f**) CNALCPA.

SEM was used to analyze the morphology of g-C_3_N_4_, ALCPA, and CNALCPA. As shown in Figure 3, g-C_3_N_4_ had a solid structure stacked by layers, while ALCPA showed a regular shape, such as a crystalline polyhedron. The combination of g-C_3_N_4_ and ALCPA led to a cluttered appearance. A large number of tiny g-C_3_N_4_ fragments covered the surface of ALCPA, and the morphology of ALCPA could no longer be observed.

**Figure 4:**
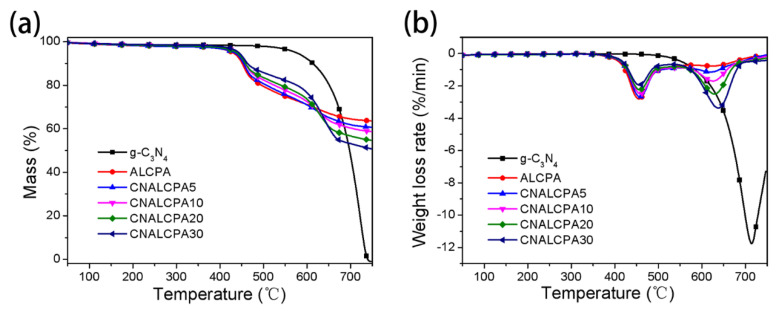
(**a**) TGA and (**b**) differential TGA (DTG) curves of g-C_3_N_4_, ALCPA, and CNALCPA.

TGA and DTG are widely used to estimate the thermal stability of materials. Figure 4 shows the TGA and DTG curves of g-C_3_N_4_, ALCPA, and CNALCPA under N_2_. Related data are in Table 3. The initial decomposition temperature and the temperature at maximal weight-loss rate are denoted as T_-5_, T_-max1_, and T_-max2_. As shown in Figure 4 and Table 3, the T_-5_ of g-C_3_N_4_ and ALCPA was 575.3 and 429.3 °C. T_-5_ continued to increase with the increasing amount of g-C_3_N_4_. T_-5_ reached 443.4 °C when the ratio of g-C_3_N_4_ to ALCPA was 30 to 100 (CNALCPA30). The initial decomposition temperature of ALCPA was nearly 200 °C higher than that of raw material CEPPA, demonstrating that ALCPA expanded the application area of CEPPA as a fire retardant.

The degradation of g-C_3_N_4_ and ALCPA was found to be one-step, but it had two steps in CNALCPA. The first degradation step was induced by the breakage of ALCPA, while the second step was responsible for the degradation of the triazine ring in g-C_3_N_4_. The triazine ring lost thermal stability over 600 °C, which could be verified by the TGA curve of g-C_3_N_4_. At 750 °C, a 63.5 wt % residue of ALCPA was obtained. However, this value continued to decrease as the content of g-C_3_N_4_ rose. The residue of CNALCPA lastly dropped from 63.5 to 50.8 wt %.

### 3.2. Fire Properties of PA6 Composites

**Figure 5:**
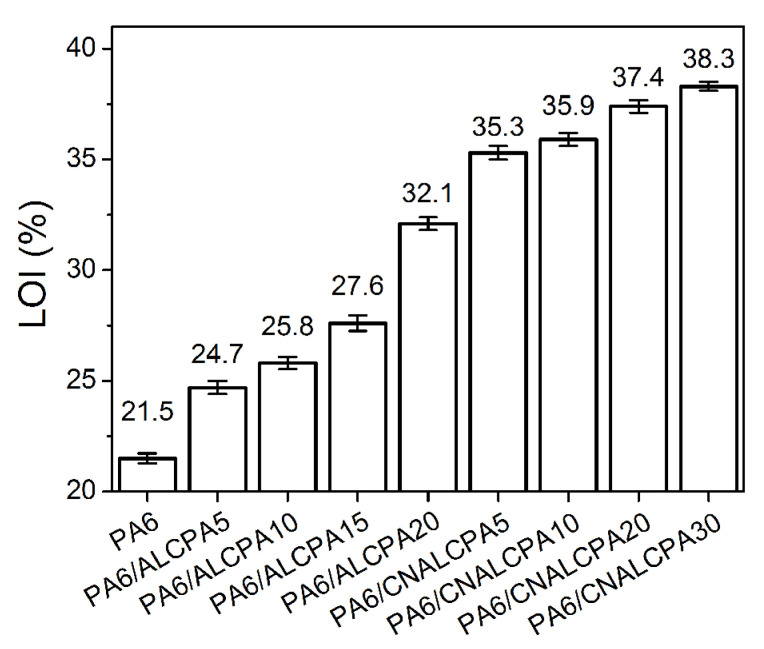
Limited-oxygen-index (LOI) values of PA6 composites.

LOI was an effective technique to characterize the flame retardancy of the materials. As shown in Figure 5, the LOI value of pure PA6 was 21.5%, indicating that PA6 is extremely flammable. The addition of a flame retardant, either ALCPA or CNALCPA, could efficiently improve flame retardancy. The LOI value was remarkably increased from 21.5% to 32.1% when 20 wt % of ALCPA was added, and further increased to 38.3% when g-C_3_N_4_ was used. The increase of LOI is usually caused by the pyrolysis products [22,23]. Non-flammable gases and some free radicals, such as PO▪ are released due to the decomposition of g-C_3_N_4_ and ALCPA which may play a role of inhibitor in gas phase. This positive phenomenon showed that ALCPA could significantly reduce the fire hazard of PA6, while the hybridization of g-C_3_N_4_ could enhance this effect.

The vertical burning test also evaluated fire properties after removing the flame. The results of the vertical burning test of the PA6 composites are in Table 4, where t_1_ was the maximal duration of the flame when the sample was first ignited for 10 s, while t_2_ was the second 10 s ignition. According to the data in Table 4, pure PA6 had no UL94 rating in either thick (3.2 mm) or thin (1.6 mm) samples. In the thin samples, along with the increasing addition of ALCPA and CNALCPA, the t_1_ and t_2_ of the PA6 composites were decreased. The PA6/ALCPA20 sample reached a V-2 UL94 rating, while PA6/CNALCPA30 reached a V-1 UL94 rating. However, the flame retardancy of thick samples was better than that of thin samples. When the same amount of ALCPA was added to samples with 3.2 mm thickness (20 wt %), its UL94 rating changed to UL94 V-1. A similar situation occurred in CNALCPA. The UL94 rating of PA6/CNALCPA30 was no longer V-1, but had become V-0. Again, these differences between ALCPA and CNALCPA showed that the hybrids could improve flame retardancy better than pure ones could.

**Figure 6:**
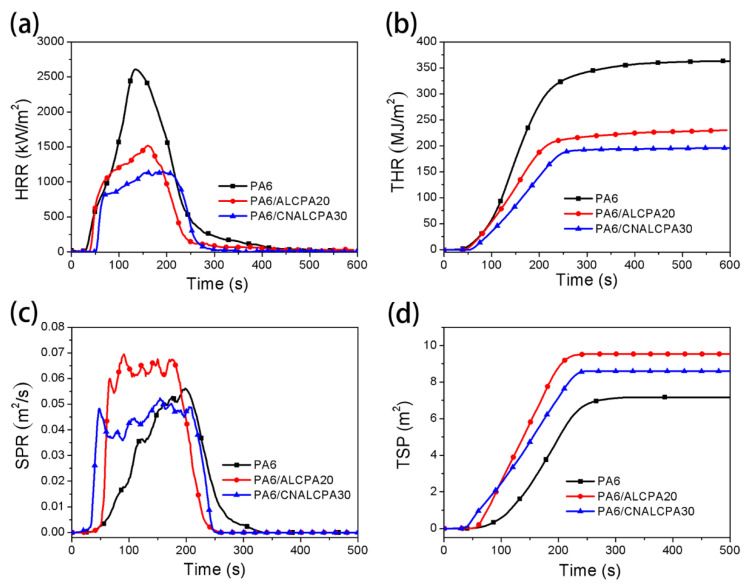
(**a**) Heat-release-rate (HRR), (**b**) total-heat-release (THR), (**c**) smoke-production-rate (SPR), and (**d**) total-smoke-production (TSP) curves of PA6 composites.

The cone-calorimeter test can provide more information about the fire hazard of materials in a real fire. The related data of time to ignition (TTI), heat-release rate (HRR), total heat release (THR), fire-performance index (FPI), smoke-production rate (SPR), total smoke production (TSP), and effective heat of combustion (EHC) are listed in Table 5 and plotted in Figure 6. As shown in Figure 6a,b, the peak heat-release rate (PHRR) of PA6/ALCPA20 and PA6/CNALCPA30 decreased from 2605.46 to 1517.59, and 1143.52 kW/m^2^, respectively, while the THR of PA6/ALCPA20 and PA6/CNALCPA30 was simultaneously decreased to 229.08 and 195.50 MJ/m^2^, 36.7% and 46.1%, respectively, lower than that of pure PA6 (363.06 MJ/m^2^). FPI was defined as the ratio of TTI to PHRR, which meant that the larger the FPI was, the longer the time to escape from fire. The addition of ALCPA and CNALCPA increased the FPI value, attesting that the flame retardancy and fire safety of PA6 composites were improved.

Smoke production is an important factor for human survival. As shown in Figure 6c,d, the peak smoke-production rate (PSPR) and TSP of PA6/ALCPA20 and PA6/CNALCPA30 were higher than those of PA6, which was probably due to the large amount of nonflammable gases produced by the decomposition of flame retardants. Furthermore, g-C_3_N_4_ have layer structure and higher onset temperature (compared with ALCPA). When ALCPA began to decompose and release smoke, g-C_3_N_4_ could still maintain its original condition and act as a barrier to keep these smoke and toxic gases in the solid phase [24,25,26]. ECH reflected the burning degree of flammable gases in fire. According to the data in Table 5, the EHC of PA6/ALCPA20 and PA6/CNALCPA30 decreased from 194.96 (PA6) to 165.06 and 124.84 MJ/kg, indicating the lower risk of a fire hazard.

### 3.3. Flame-Retardant Mechanism of ALCPA and CNALCPA

**Figure 7:**
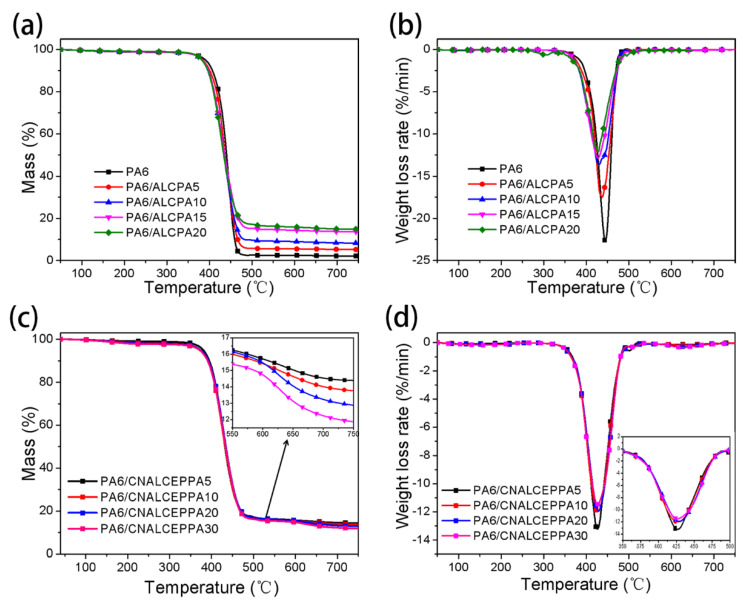
(**a**,**c**) TGA and (**b**,**d**) DTG curves of PA6 composites.

The thermal-decomposition behaviors of the composites were tested by TGA and DTG. As illustrated in Figure 7 and Table 6, it was obvious that all PA6 composites exhibited a one-step degradation process. Along with the increased addition of ALCPPA from 0 to 20 wt %, the values of T_-5_ and T_-max_ decreased, while char residue increased from 2.15 to 14.92 wt %. In addition, Figure 7b also shows that the addition of ALCPPA led to an evident reduction of weight-loss rate, suggesting that the addition of a flame retardant could delay the decomposition of PA6.

Compared with ALCPA, the hybrids resulted in a continued decline of both T_-5_ and char residue, although their addition amounts were the same. The reduced quality of char residue demonstrated that g-C_3_N_4_ played a certain role in the gas-phase mechanism. The combination of g-C_3_N_4_ and ALCPA also resulted in reduced thermal stability because g-C_3_N_4_ could catalyze the thermal degradation of ALCPA and convert it into a great amount of phosphorus- and nitrogen-containing chemicals. The decomposition of the flame retardants could absorb a lot of heat and protect PA6 from ignition. When the ratio of g-C_3_N_4_ to ALCPA increased to 20 and 30, a much weaker effect was observed, and it appeared that there was a saturation phenomenon. That’s because the barrier effect of g-C_3_N_4_ was not significant when a small amount of g-C_3_N_4_ was added. The leading role was the catalytic effect [27,28]. However, the barrier effect became more and more obvious with the increased amount of g-C_3_N_4_. The layer structure inhibited the transfer of heat and delayed the thermal decomposition of the matrix [29].

**Figure 8:**
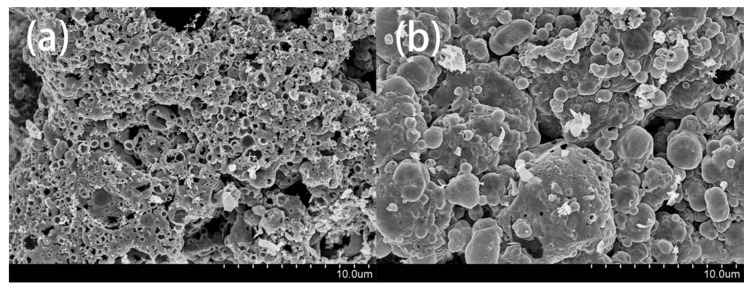
SEM images of char residue of (**a**) PA6/ALCPA20 and (**b**) PA6/CNALCPA30.

The morphology of char residue was explored, as shown in Figure 8, in order to study the flame-retardant mechanism effect that existed in the solid phase. Char residue was obtained after an LOI test. As shown in Figure 8a, many holes of different sizes appeared when 20 wt % ALCPA was incorporated in PA6, which could not effectively block the transfer of heat and oxygen. As g-C_3_N_4_ was hybridized, compact char residue without holes was gained in the PA6/CNALCPA30 sample, which could prevent the escape of flammable gases from the inner substrate to outside. The enhanced and denser char residue was more beneficial in reinforcing the anti-inflammability nature of the PA6 composites. This evidenced the solid-phase mechanism owed to the physical-barrier effect of g-C_3_N_4_.

**Figure 9:**
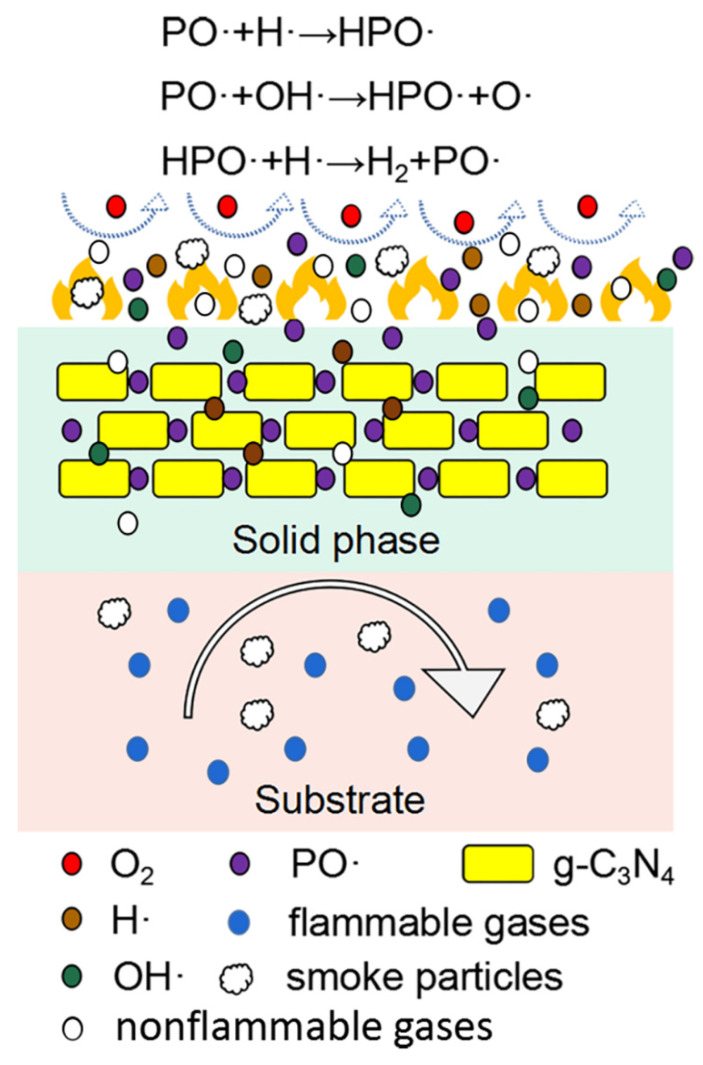
Schematic illustration for the flame-retardant mechanism of PA6/ALCAP and PA6/CNALCPA.

According to the discussions above, a proposed flame retardant mechanism for PA6/ALCPA and PA6/CNALCPA composites is illustrated in Figure 9. When the samples are exposed to the flame, g-C_3_N_4_ and metal ion can promote the char formation in the matrix and inhibit the transfer of heat and oxygen into the inner substrate. G-C_3_N_4_ can also act as a barrier in the solid phase to decrease the amount of flammable gases and smoke particles. Besides, ALCPA participates in the gas phase by generating PO· radicals, which can quench OH· and H· radicals in the flame, while g-C_3_N_4_ produces nonflammable gases to reduce the concentration of oxygen and flammable gases. The combination of solid-phase and gas-phase effect provides good fire-resistant property for PA6/ALCPA and PA6/CNALCPA samples.

### 3.4. Mechanical Properties of PA6 Composites

**Figure 10:**
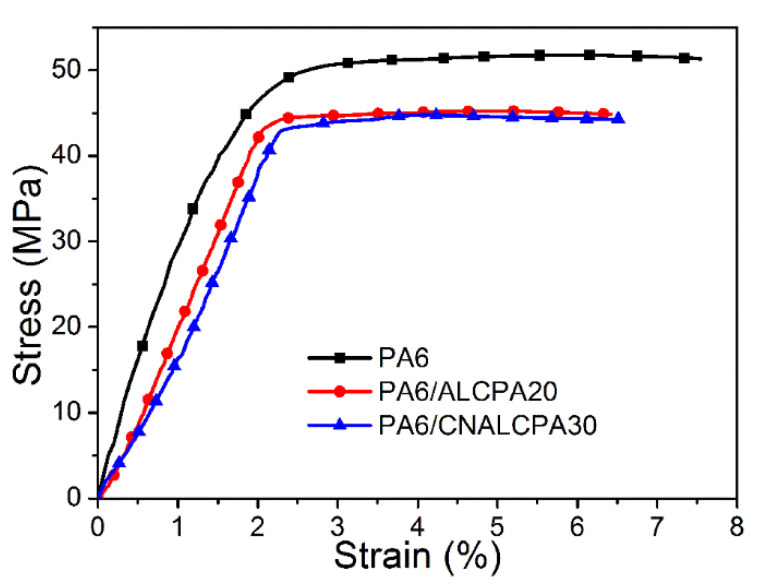
Stress-strain curves of PA6 composites.

Mechanical properties were also a key indicator if the materials could be applied in engineering. The maximal tensile strength, elongation at break and Young’s modulus of the different composites are shown in Figure 10 and recorded in Table 7. The maximal tensile strength and elongation at break of pure PA6 were 51.78 MPa, and 7.54%, respectively. However, when ALCPA and CNALCPA were incorporated into PA6, tensile strength and elongation at break had a slight decrease. Compared with pure PA6, the maximal tensile strength and elongation at break of PA6/ALCPA20 were reduced by 12.7%, and 14.7%, respectively. The hybridization of g-C_3_N_4_ did not cause more adverse effects on the mechanical properties of the composites. The performance of PA6/CNALCPA30 was very close to that of PA6/ALCPA20. Therefore, ALCPA and CNALCPA were flame retardants with good practical potential, and the PA6 composites could still maintain good mechanical properties after adding them.

**Figure 11:**
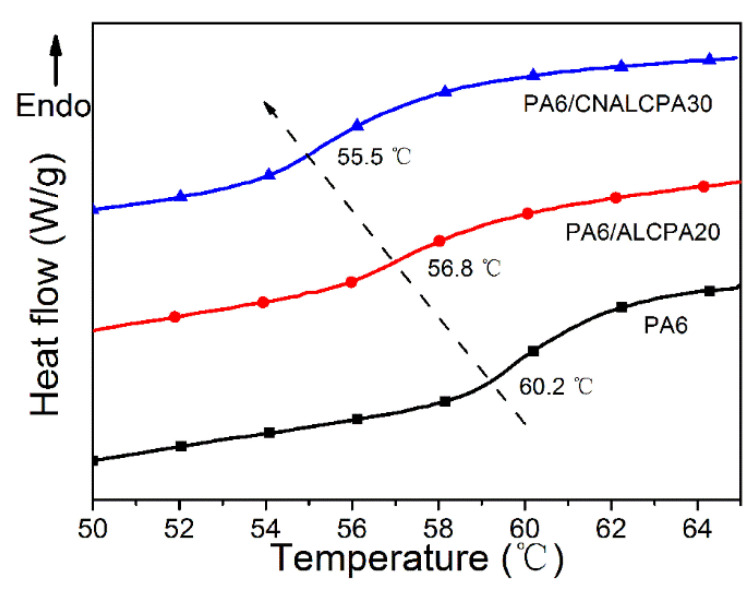
DSC curves of PA6 composites.

There is a correlation between glass-transition temperature (Tg) and the mechanical property of PA6. DSC was performed to investigate the interactions between the synthesized flame retardants and PA6 matrix, as shown in Figure 11. The Tg of pure PA6 was 60.2 °C, and it decreased when ALCPA and CNALCPA were added. Tg of PA6/ALCPA20 and PA6/CNALCPA30 were 56.8 °C, and 55.5 °C, respectively, indicating that the micro motion of PA6 molecular chain was enhanced. The possible reason is that the interactions between -P=O and -CONH- reduce the degree of crystallization of PA6 [30]. The crystallization zone limits the movement of amorphous segments, and results in a higher Tg [31].

## 4. Conclusions

The novel flame retardant ALCPA and its hybrid CNALCPA were successfully synthesized through salification and hybridization. The results of the LOI, vertical-burning, and cone-calorimeter tests showed that both ALCPA and CNALCPA displayed outstanding flame retardancy in PA6, with CNALCPA working better. After adding 20% CNALCPA30 in PA6, the LOI of PA6 could reach 38.3%, and a V-0 UL94 rating was achieved. The results of TGA and cone calorimetry also proved that ALCPA and CNALCPA could improve the fire safety of PA6, delay its decomposition, and give people more time to escape from a fire. On the one hand, the addition of g-C_3_N_4_ exerted the flame-retardant effect in the gas phase, producing nonflammable gases and reducing the concentration of oxygen and flammable gases. On the other hand, g-C_3_N_4_ could catalyze the thermal degradation of ALCPA and PA6 to form a compact char layer. Combined with the barrier effect of g-C_3_N_4_, this dense char layer can efficiently prevent flammable gases and heat from spreading.

## Figures and Tables

**Table 1:** Composition of PA6 composites.

Sample	Flame Retardant	Addition Amount (wt %)	PA6 (wt %)
PA6	/	0	100
PA6/ALCPA5	ALCPA	5	95
PA6/ALCPA10	ALCPA	10	90
PA6/ALCPA15	ALCPA	15	85
PA6/ALCPA20	ALCPA	20	80
PA6/CNALCPA5	CNALCPA5	20	80
PA6/CNALCPA10	CNALCPA10	20	80
PA6/CNALCPA20	CNALCPA20	20	80
PA6/CNALCPA30	CNALCPA30	20	80

**Table 2:** Contents of ALCPA elements.

Sample	Elements (wt %)	C	H	O	P	Al
ALCPA	Theoretical	46.96	3.91	27.83	13.47	7.83
	Actual	46.21	3.96	28.03	12.92	8.06

**Table 3:** TGA and DTG data of g-C_3_N_4_, ALCPA, and CNALCPA.

Samples	T_-5_ (°C)	T_-Max1_ (°C)	T_-Max2_ (°C)	Residue (750 °C, wt %)
g-C_3_N_4_	575.3	721.9	/	0
ALCPA	429.3	456.1	/	63.5
CNALCPA5	432.9	457.9	623.6	60.7
CNALCPA10	438.6	458.4	627.2	58.7
CNALCPA20	439.7	457.7	631.7	54.7
CNALCPA30	443.4	456.8	634.7	50.8

**Table 4:** Results of vertical burning test of PA6 composites.

Sample	1.6 mm Thickness			3.2 mm Thickness		
	t_1_/t_2_ (s)	Dripping	UL94 Rating	t_1_/t_2_ (s)	Dripping	UL94 Rating
PA6	>30/>30	BC	NR	>30/>30	BC	NR
PA6/ALCPA5	>30/>30	BC	NR	29/>30	BC	NR
PA6/ALCPA10	>30/>30	BC	NR	19.7/26.8	YES	V-2
PA6/ALCPA15	>30/>30	YES	NR	15.5/26	YES	V-2
PA6/ALCPA20	22.6/24.7	YES	V-2	11.4/22	NO	V-1
PA6/CNALCPA5	14.4/21.8	YES	V-2	9.6/10.8	NO	V-1
PA6/CNALCPA10	10.2/22	YES	V-2	6.9/10.1	NO	V-1
PA6/CNALCPA20	10.6/17.3	YES	V-1	6.2/8.9	NO	V-0
PA6/CNALCPA30	7.9/15.1	YES	V-1	4.9/7.6	NO	V-0

Note: BC, burns to clamp; NR, not rated.

**Table 5:** Results of cone-calorimeter test of PA6 composites. Note: TTI, time to ignition; PHRR, peak heat-release rate; THR, total heat release; FPI, fire-performance index; PSPR, peak smoke-production rate; TSP, total smoke production; ECH, effective heat of combustion.

Sample	TTI (s)	PHRR (kW/m^2^)	THR (MJ/m^2^)	FPI (s·m^2^/kW)	PSPR (m^2^/s)	TSP (m^2^)	ECH (MJ/kg)
PA6	47	2605.46	363.06	0.018	0.069	7.16	194.96
PA6/ALCPA20	43	1517.59	229.08	0.028	0.056	9.53	165.06
PA6/CNALCPA30	37	1143.52	195.50	0.032	0.052	8.59	124.84

**Table 6:** TGA and DTG data of PA6 composites.

Sample	T_-5_ (°C)	T_-Max_ (°C)	Residue (750 °C, wt %)
PA6	391.2	445.0	2.15
PA6/ALCPA5	385.0	436.0	5.22
PA6/ALCPA10	382.5	435.6	8.29
PA6/ALCPA15	383.6	428.8	13.66
PA6/ALCPA20	382.3	426.8	14.92
PA6/CNALCPA5	380.0	426.7	14.39
PA6/CNALCPA10	372.7	427.0	13.77
PA6/CNALCPA20	373.3	428.8	12.88
PA6/CNALCPA30	371.2	427.7	11.87

**Table 7:** Data of mechanical properties of PA6 composites.

Sample	Maximal Tensile Strength (MPa)	Elongation at Break (%)	Young’s Modulus (MPa)
PA6	51.78 ± 0.84	7.54 ± 0.20	2600.8 ± 30.3
PA6/ALCPA20	45.23 ± 0.80	6.42 ± 0.15	2056.0 ± 19.7
PA6/CNALCPA30	44.78 ± 0.68	6.51 ± 0.12	1925.1 ± 55.6
